# The Impact of Nutrition and Oral Function Exercise on among Community-Dwelling Older People

**DOI:** 10.3390/nu15071607

**Published:** 2023-03-26

**Authors:** Asuka Tani, Shinsuke Mizutani, Hiro Kishimoto, Saori Oku, Kiyomi Iyota, Tianshu Chu, Xin Liu, Haruhiko Kashiwazaki

**Affiliations:** 1 Section of Geriatric Dentistry and Perioperative Medicine in Dentistry, Division of Maxillofacial Diagnostic and Surgical Sciences, Faculty of Dental Science, Kyushu University, 3-1-1 Maidashi, Higashi-ku, Fukuoka 812-8582, Japan; atani@dent.kyushu-u.ac.jp (A.T.);; 2 OBT Research Center, Faculty of Dental Science, Kyushu University, 3-1-1 Maidashi, Higashi-ku, Fukuoka 812-8582, Japan; 3 Department of Behavior and Health Sciences, Graduate School of Human–Environment Studies, Kyushu University, 744 Motooka, Nishi-ku, Fukuoka 819-0395, Japan; 4 Faculty of Arts and Science, Kyushu University, Fukuoka, 744 Motooka, Nishi-ku, Fukuoka 819-0395, Japan

**Keywords:** food intake, oral frailty, tongue pressure, oral function, intervention study

## Abstract

Oral function (OF) decline in older people is associated with nutritional deficiencies, which increases frailty risk and the need for nursing care. We investigated whether the delivery of an oral function improvement program on a tablet device was as effective as delivery through a paper-based program. We also investigated the association between tongue pressure (TP) improvement and nutritional status at the baseline. The participants involved in the study were 26 community-dwelling older people with low TP, <30 kPa, aged ≥65 years, who were enrolled in a randomized controlled trial for a month in Itoshima City, Fukuoka, Japan. Oral and physical functions and body composition were measured at the baseline and at follow-up. Two-way analysis of variance revealed that body mass index (*p* = 0.004) increased, and maximum masticatory performance (*p* = 0.010), maximum TP (*p* = 0.035), and oral diadochokinesis /pa/ and /ka/ (*p* = 0.009 and 0.017, respectively) improved in a month. Participants with higher TP improvement showed an increased intake of animal proteins at the baseline: fish (*p* = 0.022), meat (*p* = 0.029), and egg (*p* = 0.009). OF exercises for improving TP were associated with higher animal protein intake at the baseline. This study has been registered with the UMIN Clinical Trials Registry (UMIN 000050292).

## 1. Introduction

Globally, the number of people aged 65 years was 703 million in 2019. Moreover, the aging population rate is accelerating, especially in Asian countries [1]. Maintaining the health condition of older people contributes significantly to the demand for healthcare because their general health and quality of life are declining, and older people are generally weaker than younger ones. The older health condition can be considered to have mental, social, and physical components [2]; however, a study reported that oral function deterioration, which greatly affects the physical component, is a major factor [3].

Oral function decline interferes with eating, swallowing, pronunciation, and social life. Therefore, oral function decline is associated with reduced food intake, nutritional deficiencies, and decreased conversation, which increase the risk of frailty, the need for nursing care, and mortality [4]. Consequently, an oral function assessment at an earlier stage is needed to respond to its decline immediately [5]. Oral function improvement exercises were reported to improve oral function and were shown to be effective in older people; however, the extent of improvement varies among individuals [6,7,8,9].

Oral function includes masticatory performance, occlusal pressure, tongue pressure, tongue and lip motor functions, and swallowing functions. Among them, tongue pressure was reported to be related to grip strength, which is an indicator of muscle strength [10]. Based on our previous findings, training using tools to place a load on the tongue affects physical function and body composition [6]. However, for exercises involving load placement, it is necessary to prepare different intensity tools based on the conditions of the subjects. Conversely, a past study reported oral function improvement with exercise that targeted the orbicularis oris muscle [7]. Several people, despite a lack of specific training tools, can be empowered through this approach; however, paper-based training requires the printing and distribution of booklets to participants, either directly by hand or via mail. Advances in digital material offer new opportunities for health professionals to remotely deliver and monitor exercise programs at home or in the community. These opportunities allow exercise programs to be delivered and monitored by older adults in their homes and community [11]. Consequently, IoT healthcare has great potential to improve public health [12]. However, it is unclear whether there is an equally effective approach to tablet-based exercises.

A previous study reported that individuals with oral function decline have less food intake than those with high oral function [13]. Furthermore, a trend was found that tongue pressure improved when nutritional status was enhanced in older people with general body weakness [10]. Studies have also reported that the number of foods that can be eaten increases with improved oral function [7,8]. However, no studies have investigated whether nutrient intake affects oral function improvement.

In our study, we provided oral function improvement programs via two delivery methods: tablet and paper, for individuals with low tongue pressure. This study aimed to compare the effectiveness of the two methods to ascertain whether the tablet-based program is as effective as the paper-based one. Furthermore, the aim was to investigate the association between the amount of improvement in tongue pressure as a result of programme implementation and nutrient intake at the baseline.

## 2. Materials and Methods

### 2.1. Study Design and Participants

This randomized controlled trial was conducted among residents of Itoshima City, Fukuoka Prefecture, a suburb of the metropolitan area of Kyushu Island in Japan. We invited 76 community-dwelling older people whose tongue pressure was <30 kPa based on the results of oral function tests obtained from the Itoshima Frail Study conducted in 2020. The exclusion criteria were as follows: individuals who had nursing care certification, were paralyzed, or were receiving prosthetic treatment in the dental clinic. Finally, 28 individuals were enrolled (mean age, 74.0 ± 3.7 years), and they consented to participate in the study.

This study was approved by the Institutional Review Board of Kyushu University (Application no. 202103). This study has been registered with the UMIN Clinical Trials Registry (UMIN 000050292).

### 2.2. Physical Examination

#### 2.2.1. Height, Body Composition, and Body Mass Index (BMI)

The height and body fat ratio of the body trunk and skeletal muscle mass of the trunk in addition to the weight were measured using a commercial multifrequency body composition instrument (InBody 270 analyzer, InBody Co., Ltd., Seoul, Republic of Korea) [14].

#### 2.2.2. Grip Strength

Grip strength, which was an indicator of muscle weakness, was measured using an electronic grip strength meter (Grip D TKK-5401, Takei Scientific Instruments Co., Ltd., Niigata, Japan). The second joint of the index finger was at a right angle to the grip meter, and the display was on the outside. The participants were instructed to keep their legs apart, lower their arms naturally, and grip the meter completely. The measurements were repeated two times alternating between hands. The highest value of both hands was adopted as the indicator of weakness. [15].

### 2.3. Oral Examination

Before measurements were taken, a protocol was created, and calibration was performed based on this by four well-trained dentists. The researcher who took the measurements was unaware of the allocation of the participants in the group.

#### 2.3.1. Number of Teeth and Occlusal Force

The number of teeth counted excluded tooth stumps and those with increased mobility (Miller’s classification 3), which were not functional [16]. The occlusal force was measured for 3 s of clenching of a pressure-indicating film (Dental Pre Scale II, GC Corporation, Tokyo, Japan) and was analyzed using a Bite Force Analyzer Ver. 2.1.1 (GC Corporation, Tokyo, Japan). In participants who wore dentures daily, measurements were made with the dentures in the mouth [16].

#### 2.3.2. Tongue–Lip Motor Function

The motor speed and function of the lips and anterior and posterior parts of the tongue were measured as oral diadochokinesis (ODK) [16]. The participants were instructed to pronounce each of the syllables /pa/, /ta/, and /ka/ for 5 s in succession. The automatic counter (Kenkokun Handy, Takei Scientific Instruments Co., Ltd., Niigata, Japan) automatically calculated the number of syllables per second. The syllables /pa/, /ta/, and /ka/ showed the motor speed and function of the lips, anterior tongue, and posterior tongue, respectively.

#### 2.3.3. Tongue Pressure

The maximum tongue pressure was measured using a tongue pressure-measuring instrument (TPM-02, JMS Co., Ltd., Hiroshima, Japan). This instrument measured the maximum tongue pressure when the participants pressed a balloon to the anterior palate using the tongue for a few seconds. This examination was implemented three times, and the maximum value was used for assessment [6,16].

#### 2.3.4. Masticatory Performance

A test chewing gum (Masticatory Performance Evaluating Gum XYLITOL; Lotte Co., Ltd., Tokyo, Japan; 70 × 20 × 1 mm; 30 g) containing a dyed substance that changes color from green to red when mixed with saliva was used for the test [17]. The participants were instructed to chew the gum wherever it was easy to chew at a rate of once per second for 60 s. The color of the chewed gum was evaluated on a 10-point scale immediately after chewing to minimize the effect of color change with time [18].

### 2.4. Data Collection of Other Variables

#### 2.4.1. Nutrition

A brief-type self-administered diet history questionnaire (BDHQ) [19], which was developed for the evaluation of nutritional status in the epidemiological survey, was used [20]. This questionnaire was mailed to the participants’ homes before the baseline assessment. This questionnaire assessed 58 food consumption frequencies in the last month using an ad hoc computer algorithm [21]. The participants answered the BDHQ by themselves or verbally responded to a family member when they were unable to do so. The participants completed the questionnaire and submitted it for baseline assessment. Nutrient data were analyzed using the density method to estimate intake per 1000 kcal [22].

#### 2.4.2. Cognitive Function Assessment

We assessed participants’ cognitive function using the Mini-Mental State Examination (MMSE), 2019 because a link was reported between cognitive function and oral function [23]. MMSE was performed to assess cognitive impairment. Mild cognitive impairment was defined by an MMSE score of ≤27, and suspected dementia was defined by an MMSE score of ≤23 [23].

### 2.5. Intervention

We developed a program for oral function improvement, containing six exercises to improve masticatory muscle and performance, tongue, and swallowing muscles (Table 1).

At the baseline examination, the 26 participants were randomly divided into two groups: the tablet-based group and the paper-based group. A tablet (LAVIE Tab E8HD1, NEC, Japan) with these exercises installed was distributed to the tablet-based group. A text that contained a photograph showing the video from the tablet as a still image was distributed to the paper-based group. At the baseline examination, a dentist instructed the participants on how to perform oral function exercises. Both groups were instructed to practice the preparational exercises and two of the six predetermined exercises. They were instructed to practice three times per week at home. The tablet-based group logged their implementation status and number of exercises completed on the tablet, whereas the paper-based group wrote their achievements in a notebook provided for them. The baseline assessment was followed by a 4-week program, after which participants were reassessed.

### 2.6. Sample Size

We referenced our previous intervention study [6], and the sample size was 17 in total because we assumed intervention effects of 8.46 and 4.0 kPa for the tablet- and paper-based groups, respectively. The significance level was set to <5% and the confidence level to 80%. Furthermore, 24 was considered the minimum number of participants when taking into account the number of dropouts in 1 month, which was calculated as 1.4 times the sample size.

### 2.7. Statistical Analyses

The chi-squared test for sex and the Mann–Whitney U test for other characteristics were used to compare the tablet- and paper-based groups at the baseline. A repeated-measures two-way analysis of variance (time [baseline/after intervention] × group [tablet-based group/paper-based group]) was performed to analyze the intervention effect, following the Wilcoxon signed-rank test.

To investigate the association between increased improvement in tongue pressure and nutrition intake, the participants were classified into two groups: the low improvement (LI) group and the high improvement (HI) group by a median of 1.3 kPa of the increase in tongue pressure. Moreover, we analyzed the difference in the food group intake between the two groups using the Mann–Whitney U test to determine the relationship between the extent of tongue pressure improvement and food trends.

A *p*-value of <0.05 was considered statistically significant. IBM SPSS Statistics for Windows version 28.0.1.1 (IBM Corp., Armonk, NY, USA) was used for the analyses.

## 3. Results

### 3.1. Participant’s Characteristics

**Figure 1:**
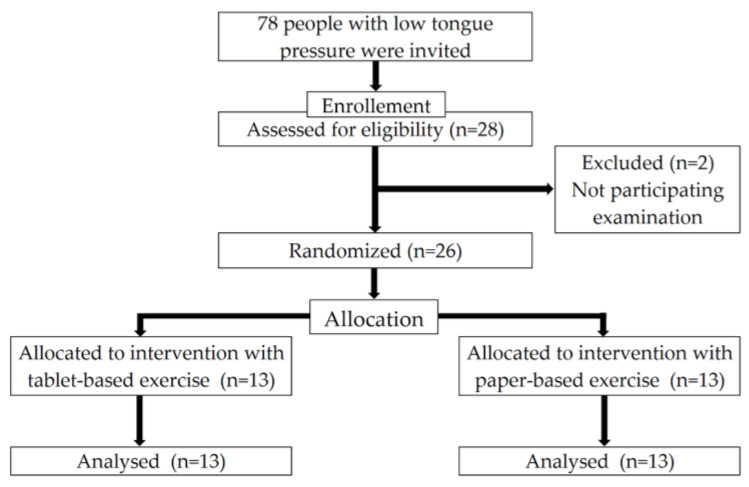
Flowchart of the enrollment of the participants with low tongue pressure. Adapted from the CONSORT2010 flow diagram.

Figure 1 shows the flowchart of this study. Initially, 78 individuals with low tongue pressure (<30 kPa) were invited to participate in the study, of which 28 consented to participate in the study. However, two participants dropped out before the baseline evaluation because of personal reasons. The participant age ranged from 75 to 81 years, with a mean of 74.0 ± 3.7 years.

As shown in Table 2, the 26 participants were distributed into 13 in the tablet-based group (five men and eight women; mean ± SD age, 74.1 ± 3.8 years) and 13 in the paper-based group (eight men and five women; 73.9 ± 3.6 years) at the baseline. The baseline examination results of the two groups were compared. No significant difference was found between the tablet- and paper-based groups in all physical and oral categories at the baseline.

### 3.2. Intervention Effect

Table 3 shows the results of the two-way analysis of variance comparing the tablet- and paper-based groups at the baseline and after intervention. The items showing interaction (intervention × between groups) included muscle mass (*p* = 0.024), skeletal muscle mass (*p* = 0.029), and SMI (*p* = 0.032). No difference was detected in the pattern of change of interaction for any other item, which indicated that the trend of change was similar between the groups.

Moreover, no significant difference was observed between the two groups, that is, the tablet group and the paper-based group, either at the baseline or at follow-up. The items in which the main effects of the intervention were observed included weight (*p* = 0.003), BMI (*p* = 0.004), maximum masticatory performance (*p* = 0.010), maximum tongue pressure (*p* = 0.035), and ODK /pa/ and /ka/ (*p* = 0.009 and 0.017, respectively).

### 3.3. Nutrient Intake

In Table 4, no significant difference in nutrient intake was found between the tablet- and paper-based groups at the baseline.

Table 5 shows the adjusted geometric means of daily nutrient intake by the increase in tongue pressure at the baseline. The participants were classified into the LI and HI groups by a median of 1.3 kPa of the increase in tongue pressure. The intake of animal protein, namely, fish (*p* = 0.022), meat (*p* = 0.029), and egg (*p* = 0.009), was significantly higher in the HI group than in the LI group. In addition, a significant difference in the intake of sugar and sweeteners was found (*p* = 0.029). Conversely, no significant difference was noted in the intake of cereals, potatoes, beans, green-yellow and other vegetables, fruits, milk products, fat and oil, and confectioneries.

## 4. Discussion

### 4.1. Effectiveness of the Home-Based Oral Training

One of the aims of this study was to improve oral functions with home-based exercises in participants with low tongue pressure (<30 kPa). In this study, the program for oral function improvement included six exercises to promote masticatory muscle and performance and tongue and swallowing muscles at home. In this study, we provided oral function improvement programs through two means—tablet and paper—and compared the effectiveness of these two methods. Our results suggested that both the approaches were equally effective. Only a few intervention studies have provided the same content of exercises by different means. The present study showed that tongue pressure and tongue–lip motor function were improved statistically significantly, although no difference in exercise effectiveness was found between the two groups.

This study showed that the proposed exercise program enhanced the maximum tongue pressure after the intervention. Moreover, the 17 participants with low tongue pressure (<30 kPa) exceeded the reference value and had normal tongue pressure after only a month. The results of our previous study [6] and those of Shirobe [7] proposed that tongue-strengthening exercises help increase tongue strength; however, the present study revealed that tongue strength can be promoted without directly loading the muscles. Although a previous study [7] reported that tongue pressure can be improved without direct training in oral function, because oral function decline is a relatively early sign of poor health [5,9], oral function status should be checked daily by implementing an oral function improvement program. Although the intervention period in this study was relatively short compared with those in previous studies [6,9,24], the period in this study was considered appropriate to observe tongue pressure improvement in participants with low tongue pressure because our previous study showed that tongue pressure improvement can be significantly improved in the first month [6].

This study also showed that masticatory performance improved after the intervention. Our previous study revealed that the physical pre-frailty status of participants was significantly associated with masticatory performance [23]. Furthermore, Hironaka et al. revealed that masticatory ability was associated with diet variety [25]. Consequently, an increase in weight and BMI after intervention might be considered the result of an improvement in oral function. Home-based exercises may prevent not only the worsening of oral function but also physical pre-frailty. Studies have reported that people with a low BMI have a higher mortality rate than those with a high BMI; thus, gaining weight is not unbeneficial [4,26].

### 4.2. Relationship between Tongue Pressure Improvement and Nutrients

This study revealed that the intake of animal proteins (fish, meat, and egg) was significantly high in the HI group. Adequate energy intake, especially proteins, was reported to be important in preventing frailty [11,27]. Previous studies have revealed the relationship between the number of remaining teeth and nutritional status [28,29] and between masticatory function and nutritional status [30,31]. Moreover, a previous study reported an association between nutritional status and poor oral function [32]. However, few studies have evaluated the relationship between tongue pressure improvement and nutrient intake. Nishi et al. also reported that oral hypofunction was significantly and independently associated with protein intake in both men and women [32]. In addition, Nagano et al. reported the need for appropriate protein intake to improve tongue strength [33]. This result might support our study. However, we have to take into account that the evaluation of nutrition intake in our study was only at the baseline, and we did not evaluate it at the follow-up. The BDHQ is a survey format that requires respondents to recall and report the foods they have consumed in the last month [21]. Because the intervention period in our study was 4 weeks, we did not implement the BDHQ. Therefore, it is unclear whether the participants’ nutritional intake changed during the 4-week oral function improvement program. Further studies need to evaluate the changes of nutrient intake and nutritional status using blood biomarkers such as concentrations of albumin, hemoglobin, and total cholesterol [34].

A few studies have reported associations between dietary advice, nutritional status, and oral function. Suzuki et al. reported that using a new pair of complete dentures and following simple dietary advice increased protein intake [35]. This suggests that dentists should not only make dentures but they should also improve nutritional status and oral function using effective methods. Although nutritional advice and the fabrication of new dentures were found to be effective, the effectiveness was only valid in the short term [36]. Therefore, effective tongue pressure improvement may be seen when people with a stable occlusal relationship are offered nutritional guidance and perform the proposed exercise program. This study also found high plant protein intake in the interventional participants [36]. The results were different from our findings, in which animal protein intake was higher than plant protein intake. In relation to this point, a previous study reported that animal protein intake was associated with the number of remaining teeth [37]. Our results revealed that high animal protein intake may be associated with participants who had many remaining teeth.

Because the estimation of nutrient intake in BDHQ was based on the Standard Tables of Food Composition in Japan, the evaluation of BDHQ may not be applicable to other races. We have to take into account that some foods, such as matcha green tea, are not included in BDHQ [38]. In addition, the validity of other nutrient and food intakes estimated by the BDHQ has not been examined among very old populations [39]. It is unclear if our findings apply to the old-old population. It is possible that answering the many questions in the BDHQ provided participants with an opportunity to re-evaluate their dietary habits, which might have had a similar effect to receiving basic nutritional guidance.

This study has some limitations. First, this intervention study might have selection bias because it was conducted in a populous area in Japan. Second, the participants were relatively healthy, and this study did not include people who could not go to the venue or go out because of malnutrition. Third, because the nutrition questionnaire used in this study was designed to examine food intake in the past month, this study did not include a nutrition survey at follow-up. Fourth, we did not examine the history of hospital visits, medical history, or medications of the participants. Therefore, the results of the physical assessment may be biased.

## 5. Conclusions

The tablet-based oral function exercises used in this study were found to improve oral functions, including tongue pressure, in community-dwelling older people. This result was comparable to that of paper-based exercises. Individuals with greater tongue-pressure improvement had a higher intake of animal proteins at the baseline.

## Figures and Tables

**Table 1:** Procedures and effects of oral function exercises.

Oral Function Exercises	Procedures of Oral Function Exercises
preparational exercise	
	This included the six exercises below. (For 3 min, the participants exercise to music.) take a deep breath raise and lower the shoulder turn your face to the right and left lean your head to the right and left widely open and tightly close your mouth fully lift the angulus oris and relax four times puff your cheek and relax fully stick your tongue out and move it up, down, left, and right take a deep breath
comprehensive exercise	
exercise for the orbicularis oris and buccal muscle	“Pushing the cheek with hands” (2 min) fully puff the cheek press the cheek with both hands 10 times while keeping the lips tightly shut to prevent cheeks from squeezing relax the cheek after pressing 10 times exercises are repeated two times “Moving air into the mouth” (2 min) puff cheek to the maximum and move the air up in the mouth, then down, left, and right for five times per set repeat the exercises two times
exercise for tongue muscle	“Moving tongue exercise” (4 min) fully protrude and retract the tongue two times and put the tongue apex to the cervical line of the maxillary central incisor when retracting the tongue move the tongue up, down, left, and right two times rotate the tongue widely and reverse the direction per set repeat these exercises two times “Making pressure using the tongue to the upper jaw” (2 min) press the tongue into the upper jaw for 2 s and release it quickly with a pop 10 times per set. Repeat these exercises three times
exercise for the swallowing muscles	“Head flexion exercise” (2 min) sit on the chair place the hand on the forehead and chin tuck rhythmically against the hand per second repeat this exercise four times in succession, keep tucking the chin with the hand resisting on the forehead for 4 s and repeat this exercise for five sets “Pushing down chin exercise” (2 min) set the thumbs of both hands to the edge of the mandible press the mandible down rhythmically once per second. In succession, keep pressing the chin while resisting the hand for 4 s. Repeat this exercise five times

**Table 2:** Comparison of variables in the two groups stratified by the oral exercise method at the baseline.

Variable	Total (*n* = 26)	Tablet Group (*n* = 13)	Paper Group (*n* = 13)	*p*-Value
age (years)	74.0 ± 3.7	74.0 ± 3.8	73.9 ± 3.6	0.762 *
sex (male)	13 (50)	5 (39)	8 (62)	0.239 ^†^
maximum hand grips (kg)	26.4 ± 6.6	24.9 ± 5.6	27.9 ± 7.3	0.204 *
weight (kg)	54.6 ± 7.3	53.2 ± 5.8	56.0 ± 8.6	0.390 *
BMI	21.3 ± 2.5	21.8 ± 2.9	20.9 ± 2.0	0.614 *
body fat mass (kg)	14.2 ± 5.2	14.9 ± 6.0	13.4 ± 4.4	0.336 *
muscle mass (kg)	38.1 ± 6.4	36.0 ± 5.9	40.2 ± 6.5	0.153 *
skeletal muscle mass (kg)	21.8 ± 4.1	20.5 ± 3.7	23.1 ± 4.2	0.153 *
SMI (kg/m^2^)	6.2 ± 0.9	6.0 ± 0.8	6.4 ± 0.9	0.264 *
number of remaining teeth	24.7 ± 6.4	24.6 ± 7.8	24.9 ± 5.0	0.614 *
maximum occlusal pressure (N)	889 ± 552	850 ± 478	928 ± 635	0.801 *
masticatory performance	8.2 ± 1.2	8.3 ± 1.3	8.2 ± 1.2	0.724 *
maximum tongue pressure (kPa)	27.8 ± 3.6	28.4 ± 3.9	27.2 ± 3.2	0.362 *
ODK /pa/ (times/s)	6.4 ± 0.6	6.4 ± 0.6	6.3 ± 0.7	0.579 *
ODK /ta/ (times/s)	6.4 ± 0.6	6.4 ± 0.6	6.4 ± 0.7	0.840 *
ODK /ka/ (times/s)	5.9 ± 0.7	6.0 ± 0.5	5.8 ± 0.9	0.448 *
MMSE score	28.4 ± 1.4	28.8 ± 1.3	28.1 ± 1.4	0.204 *

Values are shown as mean ± standard deviation or number (%). BMI, body mass index; ODK, oral diadochokinesis; MMSE, Mini-Mental State Examination; SMI, skeletal muscle mass index. * Mann–Whitney U test. ^†^ Pearson’s chi-square test.

**Table 3:** Comparison of the tablet- and paper-based groups at the baseline and follow-up.

	Tablet-Based Group (*n* = 13)			Paper-Based Group (*n* = 13)			Main Effect		Interaction (Intervention × between Groups)
							Intervention	between Groups	
Variable	Baseline	Follow-Up	*p*-Value *	Baseline	Follow-Up	*p*-Value *	*p*-Value ^†^	*p*-Value ^†^	*p*-Value ^†^
maximum hand grips (kg)	24.9 ± 5.6	24.9 ± 5.7	0.906	27.9 ± 7.3	28.9 ± 7.9	0.249	0.269	0.183	0.277
weight (kg)	53.2 ± 5.8	53.8 ± 5.8	0.003	56.0 ± 8.6	56.1 ± 8.9	0.408	0.003	0.379	0.071
BMI	21.8 ± 2.9	22.0 ± 2.9	0.004	20.9 ± 2.0	20.9 ± 2.1	0.524	0.004	0.328	0.088
body fat mass (kg)	14.9 ± 6.0	15.0 ± 6.0	0.937	13.4 ± 4.4	13.7 ± 4.6	0.307	0.369	0.502	0.468
muscle mass (kg)	36.0 ± 5.9	36.6 ± 5.4	0.058	40.2 ± 6.5	40.1 ± 6.7	0.345	0.174	0.124	0.024
skeletal muscle mass (kg)	20.5 ± 3.7	20.8 ± 3.4	0.064	23.1 ± 4.2	23.1 ± 4.3	0.322	0.205	0.128	0.029
SMI (kg/m^2^)	6.0 ± 0.8	6.1 ± 0.8	0.008	6.4 ± 0.9	6.4 ± 1.0	0.674	0.153	0.336	0.032
maximum occlusal pressure (N)	850 ± 478	950 ± 442	0.753	928 ± 635	943 ± 481	0.116	0.476	0.850	0.595
masticatory performance	8.3 ± 1.3	8.7 ± 1.2	0.059	8.2 ± 1.2	8.6 ± 1.1	0.084	0.010	0.797	0.802
maximum tongue pressure (kPa)	28.4 ± 3.9	29.4 ± 4.6	0.263	27.2 ± 3.2	29.4 ± 4.6	0.043	0.035	0.674	0.426
ODK /pa/ (times/s)	6.4 ± 0.6	6.7 ± 0.4	0.010	6.3 ± 0.7	6.5 ± 0.6	0.198	0.009	0.613	0.573
ODK /ta/ (times/s)	6.4 ± 0.6	6.5 ± 0.5	0.319	6.4 ± 0.7	6.5 ± 0.7	0.395	0.244	0.827	0.914
ODK /ka/ (times/s)	6.0 ± 0.5	6.2 ± 0.5	0.132	5.8 ± 0.9	6.1 ± 0.7	0.057	0.017	0.517	0.639
MMSE score	28.8 ± 1.3	28.8 ± 1.3	0.931	28.1 ± 1.4	28.5 ± 2.1	0.251	0.475	0.391	0.475

Values are shown as mean ± standard deviation. BMI, body mass index; ODK, oral diadochokinesis; SMI, skeletal muscle mass index; MMSE, Mini-Mental State Examination. * Wilcoxon signed-rank test, ^†^ Two-way ANOVA (intervention × between groups).

**Table 4:** Adjusted geometric means of daily food group by tablet-based and paper-based groups at the baseline.

Food Groups (g/1000 kcal)	Total (*n* = 26)	Tablet Group (*n* = 13)	Paper Group (*n* = 13)	*p*-Value *
cereals	160.0 ± 51.2	168.6 ± 45.5	151.0 ± 56.8	0.311
potatoes	28.7 ± 17.1	34.0 ± 13.8	23.4 ± 18.9	0.081
sugar and sweeteners	2.3 ± 1.1	2.2 ± 0.8	2.5 ± 1.3	1.000
beans	42.9 ± 19.7	47.2 ± 16.6	38.6 ± 22.2	0.287
green-yellow vegetables	69.8 ± 36.4	67.9 ± 33.8	71.8 ± 40.1	0.880
other vegetables	113.4 ± 44.9	104.3 ± 35.7	122.4 ± 52.4	0.511
fruits	101.4 ± 63.9	107.0 ± 54.9	95.8 ± 73.6	0.545
fish	53.0 ± 25.2	51.4 ± 24.9	54.6 ± 26.4	0.920
meat	44.7 ± 15.1	42.6 ± 16.0	46.8 ± 14.6	0.223
egg	24.2 ± 15.2	23.4 ± 17.9	24.9 ± 12.7	0.614
milk products	110.0 ± 52.4	100.9 ± 50.9	118.9 ± 54.3	0.545
fat and oil	5.4 ± 2.2	5.0 ± 2.3	5.8 ± 2.1	0.448
confectioneries	27.3 ± 16.8	31.0 ± 14.6	23.7 ± 18.6	0.204

Values are shown as mean ± standard deviation. * Mann–Whitney U test between the tablet- and paper-based groups.

**Table 5:** Adjusted geometric means of daily food group at the baseline by the increase in tongue pressure at the baseline.

Food Groups (g/1000 kcal)	Total (*n* = 26)	Increase in Tongue Pressure (kPa)		*p*-Value *
		<1.3 (*n* = 13)	≥1.3 (*n* = 13)	
cereals	160.0 ± 51.2	167.7 ± 40.3	151.9 ± 60.9	0.362
potatoes	28.7 ± 17.1	32.6 ± 18.3	24.9 ± 15.6	0.169
sugar and sweeteners	2.3 ± 1.1	1.9 ± 0.9	2.8 ± 1.1	0.029
beans	42.9 ± 19.7	40.8 ± 18.7	45.1 ± 21.2	0.960
green-yellow vegetables	69.8 ± 36.4	69.7 ± 34.6	69.9 ± 39.5	0.880
other vegetables	113.4 ± 44.9	101.6 ± 44.8	125.1 ± 43.5	0.169
fruits	101.4 ± 63.9	111.1 ± 73.5	91.7 ± 53.8	0.448
fish	53.0 ± 25.2	42.2 ± 15.9	63.8 ± 28.7	0.022
meat	44.7 ± 15.1	39.0 ± 12.8	50.4 ± 15.6	0.029
egg	24.2 ± 15.2	18.5 ± 16.4	29.8 ± 12.1	0.009
milk products	110.0 ± 52.4	112.3 ± 55.8	107.6 ± 50.9	1.000
fat and oil	5.4 ± 2.2	5.3 ± 2.2	5.6 ± 2.2	0.511
confectioneries	27.3 ± 16.8	32.9 ± 19.1	21.8 ± 12.6	0.081

Values are shown as mean ± S.D. * Mann–Whitney U test between <1.3 and ≥1.3 groups.

## Data Availability

The data that support the findings of this study are available on request from the corresponding author, S.M. The data are not publicly available due to restrictions e.g., their containing information that could compromise the privacy of research participants.
